# Large-scale system-level digitalisation initiatives in the National Health Service in England: insights from three national evaluations

**DOI:** 10.1038/s41746-026-02495-8

**Published:** 2026-03-02

**Authors:** Kathrin Cresswell, Robin Williams

**Affiliations:** 1 Professor of Digital Innovations in Health and Care, Usher Institute, The University of Edinburgh, Edinburgh, UK; 2 Professor of Social Research on Technology, Institute for the Study of Science, Technology and Innovation, The University of Edinburgh, Edinburgh, UK

**Keywords:** Business and industry, Information systems and information technology, Politics and international relations, Science, technology and society, Social sciences

## Abstract

There is a growing number of large-scale digitalisation programmes in health and care, each aiming to coordinate digital technologies across systems and organisations. This paper draws on findings from three independent evaluations of national initiatives within the English National Health Service, examining implementation, reception, and lessons for future efforts. We evaluated three national programmes collectively valued at £13 billion. We conducted 1079 interviews with implementers, frontline staff, patients, decision-makers, and vendors. We also observed 819 clinical encounters and meetings, and reviewed 2219 documents, including plans, minutes, business cases, and lessons-learned reports. Data gathered over 15 years enabled detailed analysis of temporal and contextual variation. Although programme goals differed, common themes emerged. Integrating new technologies with existing legacy systems constrained progress and demanded long-term systemic change, particularly in larger programmes involving multiple technological systems. The most significant challenges were sociotechnical. Expectations were inflated, timelines politically driven, and governance unstable, with objectives drifting over time. Conflicting priorities undermined coordination, and valuable learning was not consistently retained. National support and system-level coordination are essential for large-scale digital transformation. We propose a three-stage model: invest in infrastructure, enable shared learning, and build on these foundations to drive advanced innovation.

## Introduction

Health systems around the world are under increasing pressure to deliver more with less due to ageing populations, rising demand for services, and funding and workforce constraints^1^. In response, many countries have made significant investments in information technology to transform health and care delivery^2^. These digitalisation efforts are frequently accompanied by the aspiration to create learning health systems that can use data and digital tools to continually improve care delivery and outcomes^3^. Over time, the objectives of digital health transformation have expanded in scope, now aligned with the quintuple aim: enhancing patient experience, improving population health, reducing costs, supporting the wellbeing of care providers, and advancing equity^4^. There is growing recognition that digital transformation, not just task automation, should be the long-term goal^5^. This shift paves the way for future waves of innovation enabled by newly established digital infrastructures.

The vision for digitalisation has become increasingly broad. An enduring central element, but one that has to date only emerged partially and incompletely, is the integration of information across care settings: within hospitals across specialisms, between primary and secondary care, and across health and social care^6^. Information integration is increasingly linked to placing the patient at the centre of care, enabling more personalised, coordinated, and seamless experiences^7^.

To realise this vision, a succession of large-scale digitalisation programmes has been launched. These often span multiple providers and are delivered through regionally or nationally coordinated initiatives^8–11^. Programmes are typically driven by goals of interoperability, data sharing, and realising economies of scale. However, despite the large scope and ambition of these efforts, most are not comprehensively evaluated. As a result, opportunities to learn systematically about what works, what does not, and why have frequently been missed. This is compounded by the fact that methods for evaluating complex digital health programmes remain underdeveloped, and there is limited expertise in this area.

Evaluation plays a vital role not only in assessing outcomes and value for money but also in deepening understanding of implementation and optimisation processes, the environment in which systems are adopted, and the diverse needs of different stakeholders^12^. Historically, evaluation in health innovation has often focused on individual technology implementations rather than broader digitalisation programmes^13^. Yet to understand and improve large-scale digital change, evaluative approaches need to consider the interconnected nature of systems and the organisational ecosystems in which initiatives take place^14,15^.

The English National Health Service (NHS) offers a unique context for implementing and studying digitalisation. The degree of central administration provides considerable potential for pursuing coordinated system-wide change. However, its digitalisation journey has historically been uneven and encountered significant challenges (Box 1). Central to these difficulties are enduring tensions between national-level coordination and local autonomy in decision-making, struggles to improve data exchange across various disparate care settings, and the challenge of levelling up digital maturity across organisations with widely varying capabilities^16–18^. Additionally, there is often a disconnect between the broader policy emphasis on productivity and innovation (pushing staff to do more with less) and the day-to-day realities of delivering care on the frontline (where teams are already in constant crisis response and managing burnout).

To date, there is a glaring gap of research offering a long-term view of digitalisation across multiple large-scale programmes in the health sector. This is despite an increasing recognition that digitalisation initiatives must take a long-term view towards transformation as opposed to focusing on short-term implementation projects^19^. The gap in the evidence base limits the ability to learn from experience and improve future digitalisation strategy. In this paper, we tackle this issue by synthesising insights from three independent evaluations of national digitalisation initiatives in the English NHS over a period of 15 years. This allowed a longitudinal and contextual analysis illustrating the evolving local and health system factors associated with large scale digitalisation programmes over time. We explore how programmes were implemented, how they were received by stakeholders, and what lessons can be drawn to inform the design, execution, and evaluation of future large-scale digitalisation programmes.

This study builds on our prior independent evaluations of digital health initiatives, which provided detailed, context-specific analyses of individual programmes and their implementation challenges. While these evaluations offered depth and practical insight, they were not designed to support systematic comparison across cases or to surface recurring sociotechnical dynamics beyond individual settings. By synthesising evidence across multiple evaluations using a common analytical framework, the present study identifies cross-cutting patterns and mechanisms that shape digital health implementation and enables the development of higher-level conceptual insights that could not be derived from isolated programme analyses.

We drew on a large qualitative dataset of national digitalisation initiatives in the English NHS, spanning a period of 15 years (from 2009 to 2024). The dataset is unique due to its large volume of qualitative data gathered from a wide range of sources over extended timeframes.

Specifically, the combined dataset includes: 1079 interviews conducted with a diverse group of participants, including implementers, frontline staff, patients, decision-makers, and vendors; 819 observations of clinical encounters and meetings; 2219 documents reviewed, such as plans, minutes, business cases, and lessons-learned reports.

Data were analysed using a combined deductive–inductive coding approach, with the Technology, People, Organisation, and Macro-environment (TPOM) framework providing the deductive analytical structure and inductive codes developed to capture empirically salient issues not fully encompassed by the framework^20^. Analytical themes were identified based on their recurrence across programmes and their cross-dimensional nature, such that each theme incorporated codes spanning all TPOM dimensions.

Box 1 Digitalisation in the English National Health Service In the early 1990s, digitalisation progressed rapidly in primary care, with approximately 80% of General Practices computerised by 1993. In contrast, hospitals lagged in adopting digital tools.

During the 2000s, a more structured approach to digitalisation was introduced with the launch of the NHS Information Strategy and the subsequent establishment of the National Programme for IT (NPfIT) in 2002. This £12 billion initiative aimed to standardise digital infrastructure across the NHS through centralised procurement of a limited set of large-scale IT systems. However, while ambitious, the Programme struggled to gain traction. It was implemented during a period of institutional reform that decentralised provider structures, and this misalignment between centralised digital policy and fragmented local delivery undermined its effectiveness.

Following the NPfIT, the 2010s saw a shift towards local autonomy in digital procurement, with individual provider organisations responsible for selecting and implementing their own systems. While this allowed for more tailored solutions, it also exposed significant gaps in expertise and resources, and it resulted in a fragmented digital landscape with little standardisation and poor interoperability.

In response to this fragmentation, new national bodies such as NHS Digital and NHSX were established to provide national strategic oversight and support for digital transformation. This was followed by the publication of the NHS Long-Term Plan in 2019, which reaffirmed the central role of digital innovation in the future of health and care services.

The COVID-19 pandemic served as a major accelerator for digitalisation across the NHS, forcing rapid adoption of digital tools and remote care technologies.

Since then, the national strategy has increasingly focused on the contribution of digital innovation to productivity in care delivery and economic growth in the health technology sector, as detailed in the Artificial Intelligence Opportunities in Health and Care Action Plan 2024 and the 10 Year Health Plan for England 2025.

## Results

We provide an overview of the datasets in Table 1 and an overview of the findings in Table 2. Supplementary Table 1 provides illustrative quotes for each of the themes and sub-themes. It also maps TPOM codes, first-order concepts, second-order themes, and emerging aggregate dimensions.

**Table 1:** Dataset of the three national evaluations

	National Programme for Information Technology	Global Digital Exemplar Programme	Artificial Intelligence Lab
**Timeline**	2009–2011	2017–2020	2024
**Design**	12 longitudinal case studies	34 longitudinal case studies	Largely retrospective
**Number and type of participants**	431 interviews 174 IT managers, 140 healthcare staff, 10 programme staff, 10 managers of medical records libraries and the outpatient departments, 6 finance directors, 9 local service providers, 5 system vendors, 13 others	628 interviews (143 clinical digital leaders, 140 operational staff, 112 non-clinical digital leaders, 90 programme staff, 71 senior managers, 61 policymakers, 4 engagement leads 3 vendors, and 4 others	85 interviews 22 NHS England staff, 15 academics, 14 suppliers, 8 Department of Health staff, 6 previous AI Lab staff, 3 regulators, and 17 others
**Number of documents**	809	389	1021
**Number of observations**	590	217	12

**Table 2:** Overview of findings

Infrastructural challenges	Inflated expectations	Unstable governance, changing objectives and components over time	Multiple stakeholders with conflicting agendas	Evaluation and learning
Basic information infrastructures (Wi-Fi, EHRs) and data quality are crucial, but often an afterthought	Ambitious, politically driven implementation timelines	Changing ministers and senior managers; re-structuring of change management bodies	Change is technology-driven with limited attention to implications for adopters, system needs and priorities	Limited by a lack of baselines and consequent inability to assess outputs and impacts
Lack of forward planning	Programmes launched too quickly and ended too quickly	Tension between adhering to the overall goal and flexibility to respond to changing needs	Ongoing tension: national and local priorities	Evidence that the sharing of learning can accelerate adoption
				Limited learning between successive programmes

Each programme had a distinct focus, but all centred around an aspirational vision to orchestrate large-scale digitalisation through systemic intervention.

The NHS Care Records Service (NHS CRS), which was the backbone of the National Programme for IT (NPfIT), aimed to establish a foundational information infrastructure to promote interoperability across the health system. It included deploying centrally procured Electronic Health Record (EHR) infrastructures across all hospitals in England.

*“The central vision of the Programme is the NHS Care Records Service, which is designed to replace local NHS computer systems with more modern integrated systems and make key elements of a patient’s clinical record available electronically throughout England (e.g. NHS number, date of birth, name and address, allergies, adverse drug reactions and major treatments) so that it can be shared by all those needing to use it in the patient’s care.”* Department of Health^21^

The Global Digital Exemplar (GDE) Programme focused on implementing and optimising digital infrastructure (including EHRs and electronic prescribing systems), with an emphasis on generating learning that could be shared and scaled across the system. It was intended to achieve this by creating internationally recognised centres of excellence that would share their learning with hospitals seeking to achieve this status, thereby accelerating the implementation and adoption of EHRs.

*“A Global Digital Exemplar is an internationally recognised NHS provider delivering improvements in the quality of care, through the world-class use of digital technologies and information. Exemplars will share their learning and experiences through the creation of blueprints to enable other trusts [hospitals] to follow in their footsteps as quickly and effectively as possible*.” NHS England^22^

The third initiative, the NHS Artificial Intelligence Lab (AI Lab), was designed to stimulate the development and adoption of AI-based innovations within health and care. It invested in early- and later-stage development, implementation, and scaling of systems, and included efforts to develop national infrastructure and regulatory environments.

*“The government has invested more than £2.3 billion into Artificial Intelligence across a range of initiatives since 2014. This portfolio of investment includes…£250 million to develop the NHS AI Lab at NHSX to accelerate the safe adoption of Artificial Intelligence in health and care.”* National AI Strategy^23^

Our analysis revealed three aggregate dimensions reflecting the necessary investments in infrastructure, shared learning, and innovation for advancing the transformation of healthcare into a learning health system. These aggregate dimensions were derived from a set of second-order themes, which in turn were developed from first-order concepts generated through the coding process. For example, ‘investing in infrastructure’ was derived in recognition of the second-order theme of ‘infrastructural challenges’, which centred on the first-order concepts of ‘lack of forward planning’ and ‘basic information infrastructures and data quality’. The second-order themes structure the presentation of the findings in the remainder of this section and are presented as subheadings.

### Infrastructural challenges

Despite their differing aims and structures, these programmes encountered similar infrastructural challenges. Infrastructural challenges spanned all TPOM dimensions, including technology (e.g., dependability, data quality), people (e.g., attitudes, engagement), organisations (e.g., leadership and management) and macro environmental factors (e.g., vision, political context).

Interoperability - the ability to exchange data across different systems - emerged as one of the most significant issues provider organisations faced in their digital transformation journeys. Interoperability challenges were often compounded by limited supplier cooperation and inconsistent adherence to standards. Even when systems were technically capable of exchanging data, meaningful, clinically useful data exchanges were not always straightforward, as care processes varied across settings and specialities, leading to duplication and potential safety risks.

*“…appointments were put separately in the cardiology system and appointments were put separately in EPR [Electronic Patient Record]. And if you were lucky, they were the same. But obviously they weren’t some of the time… And it was just…data quality nightmare. Potentially unsafe.* Non-clinical Digital Leader, GDE Evaluation

However, the programmes differed in their emphasis on interoperability. In NPfIT, interoperability was a central objective underpinning its ambitious vision. By contrast, in the GDE Programme and the AI Lab, interoperability remained important but was not a defining measure of success; these initiatives were more focused on specific technologies and local change. In these cases, interoperability tended to emerge as a by-product rather than being pursued as a primary goal.

Robust information infrastructures, including reliable Wi-Fi, functional EHRs, and high-quality data, are a prerequisite for the effective implementation of new systems. Yet these foundational elements were often overlooked or not prioritised in favour of more high-profile or cutting-edge technologies. This oversight was particularly exemplified in the AI Lab evaluation, where the performance of AI tools was highly dependent on the quality and readiness of local infrastructures, many of which were not fit for purpose.

*“…something as simple as investing, yet more in the digital infrastructure...because there’s no way that we’re going to see a marked change in AI if actually they don’t have a digital infrastructure.* Policy Maker, AI Lab Evaluation

The timing of each programme shaped how innovations and infrastructures were perceived. During NPfIT, EHRs were still relatively novel, particularly in hospitals, as foundational infrastructures. By the time of the GDE Programme, some hospitals were re-implementing these systems, and the focus had shifted to optimising and effectively using upgraded infrastructure. When the AI Lab was launched, EHRs had become commonplace, while AI was reaching the peak of its hype cycle. At this stage, EHRs were no longer viewed as novel technologies but as established components of the healthcare infrastructure. Once embedded, hospitals became increasingly reliant on these systems.

Development of an integrated information infrastructure calls for long-term vision and planned investment over decades. However, pursuing this vision was frustrated by a system that did not support long term planning. Key challenges included: annualised budgets, change funded through a succession of episodic programmes, strategic decision makers wanting to fund new programmes in preference to extending old programmes, leadership turnover and rapidly changing policy priorities. As a result, investments were inconsistent, leading to an incomplete, fragmented infrastructure that struggled to support efforts to promote data sharing and innovation. The underlying challenges did not change over time and continue to influence the delivery of ambitious change programmes in the health service to this day.

Although programmes faced some common technical challenges, most challenges were sociotechnical. We will discuss these in turn.

### Evaluation and learning

Evaluation was an inductive theme that emerged alongside the TPOM dimensions. A common issue across programmes was the lack of good-quality baseline data. This made it difficult for programme managers and organisational stakeholders to assess outcomes, evidence progress and justify future investment.

*“… what I understand is that the initial business case sort of promised a lot, but it didn’t lead into a set of creation of baseline metrics.”* Manager, AI Lab Evaluation

Qualitative evidence of progress was much easier to obtain but difficult to measure and therefore received limited attention from high-level decision makers. For example, the GDE Programme provided strong evidence that learning can accelerate adoption and improve implementation outcomes. We observed two levels of learning. Firstly, learning within a programme that allowed local adaptation and then the application of that experience more generally. This was facilitated through cross-organisational networks involving similar technologies, populations, or professional groups. Secondly, there were some efforts to carry lessons and experience forward across multiple programmes.

*“We spent, you know, a two-hour session understanding, with the right people in the room, what (their GDE partner) did… it’s taken them five years to develop it and we did it in, you know, in one year.”* Clinical Digital Leader, GDE Evaluation

While learning emerged as one of the most valuable outcomes of all three programmes, it proved difficult to evaluate and measure. There was limited evidence of structured learning being captured and applied either within individual programmes or across different initiatives. In many cases, measurement efforts focused on what was most easily quantifiable.

*“This whole thing around benefits realisation is really a bugbear of mine. Because if they want a good qualitative evaluation, then we need to do that separately rather than look at it from a milestone perspective and also give it time to embed to see whether it benefits people”.* Clinical Digital Leader, GDE Evaluation

Across multiple programme evaluations, lessons identified were often strikingly similar, yet they were rarely applied in subsequent practice. As a result, there was a recurring loss of organisational memory, with programmes repeatedly facing the same challenges without learning from past efforts.

We also observed substantial variation across programmes in how evaluation and learning were approached. Although NPfIT included a multi-million-pound evaluation programme, little was done to integrate emerging evidence into delivery, and the programme’s public demise further limited lesson-sharing as attention shifted to cost recovery and blame. In contrast, the GDE Programme sought to learn from past shortcomings by introducing mechanisms such as funding gates, in which central support was conditional on demonstrated local progress, and by incorporating evaluation findings into ongoing strategy. The AI Lab commissioned a largely retrospective evaluation that yielded valuable lessons but offered limited opportunities for formative feedback during delivery.

### Unstable governance, and changing objectives and components over time

This theme spanned all TPOM dimensions, including technology (e.g., usability), people (e.g., work processes), organisations (e.g., leadership and management) and macro environmental factors (e.g., economic considerations and incentives). The multi-agency nature of programmes (including NHS England, the Department of Health and Social Care, and in some instances newly established delivery bodies such as NHSX and NHS Connecting for Health) offered important benefits, not just in leveraging larger investments but also in strengthening the link between policy and delivery. However, it also meant that initiatives were adversely affected by unstable governance, competing priorities, values and methods of different sponsors. Ministerial turnover, periodic organisational restructuring of delivery bodies and other external factors such as the COVID-19 pandemic (in the GDE Programme and the AI Lab), further contributed to this instability.

*“It’s a multiagency programme and by their very nature there’s always a little ambiguity in the governance, but our governance seems to have drifted in change with the changing responsibilities of different bodies during the lifetime of the Programme. And that’s not unusual but it has been particularly disruptive I think in this programme. Even changes at the top of the shop, changes of Secretary of State have an impact on our programme, because the ultimate goal or importance that the Programme is given, changes with that change of direction.”* Engagement Lead, GDE Evaluation

Some flexibility in programme direction and delivery is desirable as well as necessary. However, balancing the need to remain aligned with overarching programme goals, while also retaining the flexibility to respond to evolving priorities and circumstances, presented a recurring challenge.

In this respect, the presence of central delivery bodies in both NPfIT and the GDE Programme helped provide a clearer sense of direction and a more integrated strategy. The AI Lab, by contrast, lacked such central coordination and progress was further hampered by the COVID-19 pandemic, which intensified these challenges. The pandemic had only a marginal impact on the GDE Programme, as most of its activities were already nearing completion.

### Multiple stakeholders with conflicting agendas

The multiple stakeholders with conflicting agendas theme spanned all TPOM dimension,s including technology (e.g., adaptability/flexibility), people (e.g., engagement), organisations (e.g., leadership and management) and macro environmental factors (e.g., political context). Programmes involved multiple stakeholders with differing, and at times conflicting, agendas. They were frequently technology-led, with limited consideration of the socio-organisational aspects of change and the specific needs and priorities of the wider system. This imbalance created a risk of stakeholder disillusionment, particularly when the introduced technologies failed to meet expectations or to deliver perceived value, despite being promoted as solutions that would simplify and enhance clinical and operational workflows.

*“I think the main problem with the Programme is the expectation that it’s a little bit like going round installing three thousand copies of Microsoft Word and the following day everybody has got a Word processor. It just is not that simple”.* Healthcare Manager, NHS CRS Evaluation

The temporal context also shaped these developments. When NPfIT was launched, implementers had limited appreciation of the organisational transformation required alongside technological change. By the time of the GDE Programme, there was growing recognition that such dimensions were critical, and this understanding was reinforced and professionalised through parallel initiatives. Although this awareness remained when the AI Lab was established, the discourse became more technology-driven, as AI increasingly dominated the political and economic agenda.

There was further a sustained tension between national and local priorities which often complicated implementation and hindered sustained progress. While national-level buy-in and leadership were critical to initiating and resourcing digital programmes, successful adoption depended heavily on local engagement and ownership.

*“As a [hospital] vision, they talk about figures, ease of communication and all the buzz words. They put no thought into the nitty gritty and how clinical teams will use it and so with regard to the long-term vision, I can see what they see is a lovely neat system where we are all using computers. That is a very superficial view. To run [system] properly and the success of [system] depends on the clinicians. They need to come down a few layers and get people working with the clinicians from day one”.* Healthcare Professional, NHS CRS Evaluation

Some programmes were more effective than others in bridging this gap, particularly where strong links existed between local organisations and the centre to facilitate the exchange of information. In the GDE Programme, for example, newly created Engagement Lead roles played this function effectively. By contrast, central delivery bodies such as NHS Connecting for Health, which oversaw NPfIT, struggled to achieve the same effect.

The combination of strong central oversight and a lack of flexibility and engagement sometimes hindered local innovation. For example, while frameworks such as the Healthcare Information and Management Systems Society (HIMSS) Electronic Medical Record Adoption Model (EMRAM) offered a structured roadmap for achieving digital maturity in the GDE Programme, it was often experienced as generic and prescriptive, limiting the capacity of organisations to develop tailored solutions that met their unique needs.

*“I think pursuit of HIMSS has hindered us in our development. I think it’s delayed some things that we wanted to do and could have done sooner because we’ve had to focus on HIMSS”.* Clinical Digital Leader, GDE Programme

### Inflated expectations

The inflated expectations theme spanned all TPOM dimensions, including technology (e.g., performance), people (e.g., attitudes and expectations), organisations (e.g., leadership and management) and macro environmental factors (e.g. political context). Programmes were shaped by inflated expectations, often driven by ambitious, politically motivated implementation timelines that prioritised rapid delivery. As a result, those charged with delivery struggled to manage the expectations of frontline staff, who anticipated that adoption would be smooth and would fairly rapidly deliver improvements.

*“…the difficulty has been managing expectations. I think the end users feel they have been lulled into a false sense of security so when we get this system, it was going to be doing all this and that, we were going to get it soon, the implication was that it would be fairly easy to implement, and it hasn’t been”.* IT Manager, GDE Evaluation

However, some programmes and settings encountered greater difficulties than others, particularly where technologies were perceived as externally imposed, required major infrastructural upgrades with long lead times before benefits could be realised, or offered limited usability for frontline staff. This was especially evident in NPfIT, where one EHR system was deployed while still under development and was widely criticised for disrupting established workflows. Notably, this system is only now being phased out of the NHS. By contrast, digital exemplar hospitals in the GDE Programme often had planned system implementations on their roadmap and invested significant effort in adapting them to local contexts and engaging clinical stakeholders to support adoption. Many of these systems had already proven successful in other settings, which further facilitated uptake.

Ministerial announcements of imminent progress generated intense pressure to demonstrate rapid improvements. This did not align with the complex and time-consuming nature of digital transformation, which typically unfolds over a period of years or even decades. Those delivering programmes spent significant time and resources seeking to evidence progress and held those delivering local change to account to strict implementation deadlines. Given that outcomes would typically emerge more slowly than programme lifetimes, they often sought to evaluate proxy measures (e.g. inputs rather than outcomes). However, these proxies might not accurately predict eventual desired outcomes.

*“You know, they genuinely just want to measure something. They don’t really care what it is”.* Policy Maker, AI Lab Evaluation

Overall, programmes were launched prematurely, with limited time for baseline assessments or thorough planning. They also ended too soon, before long-term benefits could be fully realised or demonstrated within the constrained political or funding cycles.

However, the technologies introduced across programmes varied, and so did their pathways to impact. EHRs implemented and optimised under NPfIT and the GDE Programme required long timelines before delivering measurable benefits, as they depended on large-scale organisational transformation. By contrast, many of the technologies supported by the AI Lab offered shorter routes to impact, often through automation and more targeted applications.

## Discussion

Our analysis provides longitudinal and contextual insights into evolving organisational and health system factors associated with large-scale digitalisation programmes over time. While the programmes studied differed in their specific aims and focus areas, several common themes emerged. A recurring challenge across all initiatives was the integration of new technologies with existing information infrastructures. These underpinned but also often limited the scope for further digital development, connection of diverse systems, and integration of advanced digital capabilities.

However, the most substantial challenges were sociotechnical rather than technological. We observed a consistent pattern of inflated expectations and politically driven implementation timelines that were not aligned with the realities of frontline delivery of care. Governance structures were unstable, and as programmes evolved, goals and components frequently shifted, creating uncertainty and inhibiting progress. Although learning was one of the key benefits of programmes, valuable insights gained were not consistently curated and applied.

National support and system-wide intervention play a critical role in enabling effective digitalisation in healthcare. They are particularly valuable in promoting interoperability, scalability, and sustainability, as well as in facilitating the sharing of lessons and learning across organisations and settings. Systemic intervention is also critical for building and strengthening robust information infrastructures, which are essential for long-term progress.

**Fig. 1:**
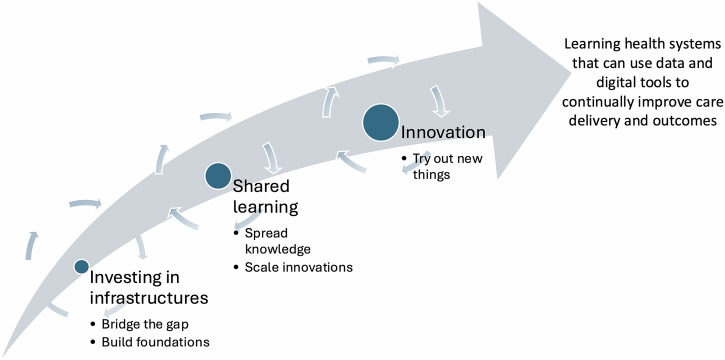
Innovation, Learning, and Infrastructure for Advancing Systemic Digitalisation (ILIAD Model, created with PowerPoint).

Building on this work, we propose a new model for conceptualising digitalisation enabled by systemic policy change in healthcare: the ILIAD Model, which stands for *Innovation, Learning, and Infrastructure for Advancing Systemic Digitalisation* (Fig. 1). This model offers a strategic, long-term approach to digitalisation at the system level, structured around three interconnected components. It is designed to inform strategic decision makers when planning and designing large-scale digitalisation programmes in health service settings.

The model includes three stages to reflect a necessary sequence: first investing in infrastructures, then enabling shared learning, and finally investing in innovation. Infrastructural foundations are a prerequisite for learning and innovation, and while all three components are equally important, progress is unlikely without this base.

The first component addresses the need to strengthen basic digital infrastructures, especially in less digitally mature settings. This involves investing in data quality, system interoperability, and foundational capabilities to level up across the system. Such infrastructure is essential not only for supporting immediate improvements but also for laying the groundwork for future innovation.

The second component emphasises learning, particularly through the creation of structured relationships between high-performing innovators and those organisations that aspire to reach similar levels of digital maturity. This peer-to-peer model echoes the successful dynamic of the GDE Programme, where “fast followers” were paired with exemplar sites to accelerate shared learning and spread innovation. Establishing and coordinating these learning networks can facilitate scaling of effective practices and technologies across the system.

The third component focuses on further enhancing innovation in digitally mature settings. These environments, often linked to academic institutions and underpinned by robust information infrastructures, are well-positioned to experiment with new technologies and approaches. They typically have the resources, culture, and capacity to support rapid innovation, making them ideal testbeds for pioneering digital innovations.

The ILIAD Model offers a balanced multi-stranded strategy, supporting innovation in leading sites, enabling system-wide learning, and ensuring future-oriented infrastructure development. By aligning with both political and operational goals, the model addresses the need for demonstrating progress, supporting innovators in pushing boundaries, and building the essential foundations for a learning-oriented health system.

Together, the ILIAD model and the findings from this study contribute to the existing literature and practice in five key ways, which are outlined below.

First, the issue of scale emerged as a critical factor in our work. All programmes attempted to digitalise a national health system for a population of ~57 million. There remains considerable uncertainty about the optimal scale for digitalisation programmes, whether national, regional, or local. Our findings suggest that large-scale digitalisation efforts may succeed when using relatively simple, standardised applications, but are more likely to struggle with complex functions that resist standardisation, as these can become too unwieldy for organisations to adopt and sustain. In addition, building from simple to complex functionality, while allowing room for modular expansion, appears to be a more viable path for long-term digital transformation than ambitious “big bang” implementations where all functionality is launched simultaneously^24^. A modular and iterative strategy is therefore most likely to succeed, including a nationally standardised element “loosely coupled” with regional level implementation^25^.

Second, standardisation has been consistently identified as a key enabler of interoperability, particularly in large programmes^26–28^. However, our research reinforces the risk that rigid standardisation may fail to accommodate local variation and evolving system needs, leading to the abandonment of systems that do not meet frontline or contextual requirements. As such, digitalisation strategies must strike a careful balance between achieving interoperability and allowing local flexibility and adaptability^29^. As more digital capabilities are introduced and built on existing information infrastructures, there is an increased risk that these will become difficult and expensive to manage, leading to fragmentation rather than the intended cohesion.

Third, our findings illustrate the challenge of orchestrating systemic policy interventions in healthcare through large systems change, where change occurs at both system level and within organisational environments^30^. The difficulty for those orchestrating change is that the health system is a complex adaptive system^31^, where various interconnected actors, technologies, and organisational dynamics evolve continuously. In such environments, change cannot simply be imposed. It must be coordinated, iteratively developed, and responsive to emergent needs and contexts. Overly directive national strategies may lead to resistance and risk stifling local innovation and adaptability, while a purely “bottom-up” approach may struggle to achieve coherence and scale.

Fourth, we have shown that learning, both within and across programmes, plays a vital role in navigating these tensions. However, it is extremely difficult to aggregate learning from individual organisational settings and apply it meaningfully elsewhere, particularly at scale and in complex public service environments^32,33^. While learning has the potential to contribute to policy and system change, it is often inhibited by institutional and political dynamics^34^. We have shown that changes in government, shifting ministerial priorities, and limited attention to system-wide needs can undermine efforts to embed and act on learning. In line with this, evidence shows that policy decisions are frequently shaped by individual and organisational interests, which may conflict with broader learning goals^35,36^.

Fifth, a central dilemma in large-scale digital health transformation lies in the tension between shared objectives and conflicting needs at the national and local levels. There is therefore a need to take a multilevel view of value co-creation^37^. National policymakers are likely to prioritise cost control, system-wide efficiency, and population-level outcomes. In contrast, healthcare organisations, focus on improving the efficiency and performance of their own services and responding to the needs of their local populations. These differing priorities can create friction, particularly when national strategies require significant integration efforts that may not immediately benefit individual organisations. In such cases, organisations may be disincentivised from investing in shared data systems as they have differing priorities and histories, even though collective action would produce benefits at the system level. This reflects a classic collective action problem, where the overall system gains only if all parties participate, but no single organisation may be willing to bear the costs alone^38^.

Despite offering the first longitudinal exploration of a series of national digitalisation programmes in healthcare, this study has some limitations. The dataset underpinning the analysis is unique and comprises a large volume of qualitative data collected from multiple sources over extended time periods, and the evaluations were undertaken by a stable research team using an evolving but shared theoretical framework. This enabled learning to be carried across programmes and contexts. However, the richness of the dataset also created analytical challenges. In prioritising the most prominent and recurrent themes, some nuance may have been lost, and the use of a single overarching coding framework, while enabling comparative analysis, may have constrained the identification of more emergent or contradictory interpretations. To mitigate this, we included illustrative quotations reflecting diversity within themes (Supplementary Table 1) and explicitly highlighted divergent views where they arose in the Results section. In addition, the formative nature of the evaluations meant that the research team became active participants in shaping the programmes under study, and the evaluation therefore formed part of the intervention itself. This was addressed through reflexive documentation and discussion, transparent reporting, and systematic triangulation across data sources. Finally, although the original study designs included a quantitative component, this could not be consistently implemented across all programmes, limiting cross-programme comparison. Substantial variation between settings, the evolving nature of the interventions, the absence of robust baseline data, and the relatively short duration of the programmes further constrained assessment of longer-term outcomes and sustainability. Nevertheless, this work provides valuable insights into the orchestration of large-scale digital change and the dynamics of systemic intervention in healthcare settings.

We here provide an integrated sociotechnical account of how national digital transformation in the NHS is shaped by the cumulative and interacting effects of infrastructure, governance and stakeholder dynamics, rather than by isolated “critical success factors”. By examining NPfIT, the GDE Programme and the AI Lab together, we show that successive programmes foregrounded different priorities (i.e. foundations, learning and innovation) while neglecting others, and that sustainable system change requires sustained and balanced investment across all three^39–44^. In a health and social care context, where integrated care and cross-setting pathways make robust, interoperable information infrastructures uniquely critical, national programmes can catalyse innovation and adoption, but only if they are underpinned by a long-term, cross-political vision and effective mechanisms for evaluation and institutional learning. Digital transformation should therefore be understood not as a sequence of discrete initiatives, but as an ongoing, adaptive sociotechnical process that must continuously align infrastructure, innovation and learning while actively managing competing priorities across policy, organisational and staff levels.

## Methods

Detailed methods for each of the evaluations are reported in published papers^45–47^. We here present an overview of methods highlighting similarities and differences and the comparative element across projects. Evaluations included in this analysis were national evaluations of:

- The NHS CRS, a national attempt to implement Electronic Health Records across English hospitals as part of NPfIT
- The GDE Programme, a national initiative to support selected digitally advanced hospitals to become international exemplars and create a national learning ecosystem
- The NHS AI Lab, a national programme to facilitate the safe deployment of AI in the NHS.

We provide a description of each of the projects illustrating why they constitute mega-projects in Table 3.

**Table 3:** Case description of the three national programmes studied

	National Programme for Information Technology	Global Digital Exemplar Programme	Artificial Intelligence Lab
**Duration**	2002/2003 - 2011	2017 - 2021	2019-2024
**Funding**	£12.7 billion	£302 million	£143.5 million
**Vision**	Aimed at transforming the NHS through IT. Its delivery was planned to establish a national electronic infrastructure, including a broadband network (N3), electronic prescription and appointment booking services, and a “cradle to grave” Electronic Health Record (EHR) for every patient by 2010.	Aimed to drive digitally enabled transformation in selected NHS provider organisations, bringing them to an international standard, and to foster a national learning ecosystem	Core vision to accelerate the safe adoption of AI in health and social care settings.
**Components**	The Spine (central national database and messaging service). Choose and Book (electronic referrals). Electronic Prescription Service (EPS). Picture Archiving and Communication Systems (PACS) for imaging. Ambition for Electronic Patient Records (EPRs) across all hospitals (focus of our evaluation).	Exemplar provider organisations (funded to reach international digital standards). Fast Follower provider organisations (paired with exemplars to adopt and adapt blueprints). Blueprints (digital implementation guides for wider replication). Digital maturity assessments (frameworks for benchmarking progress).	AI Skunkworks (rapid proof-of-concept projects, open-source outputs). AI in Health and Care Awards (funding for promising AI solutions to be tested and scaled). AI Deployment/ Diagnostic Funds (especially in imaging and diagnostics). AI Communities of Practice (guidance, case studies, resources). Ethics, Regulation, and Governance workstreams (safe and responsible adoption).
**Delivery**	Connecting for Health (CfH) was created in 2005 as the delivery arm within the Department of Health and took operational responsibility for NPfIT. The NHS signed contracts with private sector companies, each covering a large geographic area delivering services.	Managed by NHS England (digital transformation team). Funding was provided centrally, but settings were responsible for local delivery.	The AI Lab worked across national, regional, and local levels, but with a distributed structure. Some aspects of the programme operated centrally (e.g. setting strategy, national resources) and others were more localised proofs of concept or implementations. It was delivered jointly by NHS England and the Department of Health and Social Care.
**Scale**	National	National	National
**Ministerial backing**	Launched in 2002 under Prime Minister Tony Blair with strong backing from the Department of Health, led by Secretary of State for Health Alan Milburn and later John Reid; Blair himself personally championed it as a flagship modernisation project.	Announced in 2016 with support from Secretary of State for Health Jeremy Hunt, who made digital transformation a central plank of his patient safety and NHS modernisation agenda.	Launched in 2019 with ministerial support from Secretary of State for Health and Social Care Matt Hancock, who strongly advocated for AI and innovation as central to the NHS Long Term Plan.
**Public visibility**	Highly visible, widely covered in the media as the world’s biggest civilian IT project, but became notorious for delays and cost overruns.	Lower public profile, mainly recognised within NHS and health technology circles as a professional digital transformation initiative.	Moderate visibility, often featured in government announcements and health innovation news, framed around AI’s potential for patient care.

All three evaluations were largely qualitative, with two also having small quantitative elements (NHS CRS and AI Lab evaluations). The quantitative work was limited in scope and robustness due to the lack of available meaningful baseline and comparative data. We also faced challenges with benefit attribution and the difficulty of quantifying long-term, systemic changes during short-term evaluation lifecycles. We therefore did not include these elements in the current paper.

### Sampling

We used purposeful strategic sampling to select participants, organisations, and data sources to ensure representation of a diversity of viewpoints and contexts. Gatekeepers at policy and organisational levels helped establish initial contacts and gain access to participants and documents. Based on these engagements, we employed snowball sampling to identify additional participants, explicitly looking for discordant viewpoints.

We sampled individual participants from technology, frontline, organisational and macro-environmental settings (those outside participating organisations e.g. policy, strategy and supply) to include a range of perspectives. Individuals were specifically chosen to represent a range of demographics, degrees of seniority and professional backgrounds. The AI Lab evaluation sampled individuals and documents related to various components of the AI Lab. It did not explicitly use a case study-based approach to study implementing organisations.

In evaluations where we drew on organisational case studies as an approach (NHS CRS and GDE), we sampled provider organisations to maximise variation across a range of geographical settings, with various EHR systems, and with diverse funding profiles and sizes. The GDE evaluation included a core set of 12 in-depth hospital case studies and a broader sample of 24 hospital sites for more limited data collection. The NHS CRS evaluation included data from 12 hospitals.

### Data collection

All three evaluations relied heavily on semi-structured interviews as the primary data collection method. Interviews explored participants’ experiences, perspectives, challenges, and lessons learned. They were, where possible and relevant, conducted longitudinally to explore experiences over time before, during and after implementations of relevant systems and strategies (GDE and NHS CRS evaluations). Common themes explored across evaluations included technological, organisational and strategic contexts; expectations, attitudes and (where relevant) experiences and reflections of the digitalisation initiative; and implications for strategy and future initiatives. Across our projects, we focused on exploring diverse stakeholder perspectives and how these shaped observed progress, including unexpected consequences. A sociotechnical lens guided our approach, grounded in the core assumption that technological and social dimensions are deeply intertwined and must be examined together when studying digitalisation^48^. Interviews were audio-recorded, anonymised and transcribed.

All evaluations also included documentary analysis of relevant reports, minutes of planning and review meetings, strategies, and other programme-related materials. These documents were shared by commissioners and gatekeepers. They provided important contextual background and insights into the implementation, adoption, and optimisation processes as planned and as viewed at a particular point in time.

We also conducted non-participant observations of individual care activities (in GDE and NHS CRS evaluations), strategic and planning meetings and workshops. During these we took fieldnotes focusing on exploring how different stakeholders interacted and how strategies were conceived and implemented in practice.

The three evaluations were conducted between 2009 and 2024. They varied in length from three years (GDE and NHS CRS evaluations) to eight months (AI Lab evaluation). Data were collected longitudinally in the GDE and NHS CRS evaluations, which tracked developments over time through multiple site visits. The AI Lab evaluation was primarily retrospective, exploring activities that had already occurred.

### Analysis

All three evaluations drew on converging sociotechnical analysis approaches to analyse the complex interplay between technology, people, organisations, and the wider environment. The NHS CRS evaluation contributed to developing the Technology, People, Organisations, and Macroenvironmental factors (TPOM) framework, which was then validated and tested in the other two evaluations^20^. We used a combination of deductive and inductive approaches to coding and analysis guided by sociotechnical frameworks but allowing new themes to emerge from the data. Across evaluations, we used NVivo to code extracts. Lead researchers began by analysing case data before we made cross-case comparisons, extracting commonalities and differences across cases and stakeholder groups.

Analysis focused on extracting experiences (positive and negative), perceived outcomes and challenges, as well as identified lessons. Findings were discussed and validated within the research team through designated analysis workshops. Emerging findings were used to inform subsequent data collection and were fed back to commissioners (the formative element of the evaluations). The analysis across evaluations aimed to extract transferable lessons for future digital transformation initiatives.

To identify cross-evaluation themes, the authors (who have been involved in data collection and analysis of all three evaluations) conducted a secondary analysis of the data sets. This secondary analysis focused on exploring technological, social, organisational and wider system factors associated with programme implementation and adoption over time. We identified commonalities and differences across these dimensions and highlighted tensions and trade-offs among stakeholder groups. This was achieved by initially coding the data sources according to TPOM categories and sub-categories (see Table 4).

**Table 4:** High-level coding framework

TPOM Dimension	Dimensions underpinning the TPOM factors in Cresswell 2020
**Technology**	Technological systems, functionality, interoperability, reliability
**People**	Stakeholders, roles, experiences, engagement, skills
**Organisation**	Structures, processes, resources, leadership, governance
**Macro-environment**	External influences, policy, regulation, wider system context

Commonalities across the dimensions were identified through examining high-level codes across programmes. We did allow for a degree of variability within these, as otherwise the analysis would have been too complex (e.g. system functionality inevitably varied across settings and programmes). Areas of greatest convergence (i.e. those that came up across all three evaluations repeatedly as strong themes) were then included in the narrative presented in the Results section. Tensions and trade-offs across the dimensions were identified through examining findings across programmes, across dimensions (e.g. when data did not fit in neatly within one code or explicitly contradicted it), and across time periods.

To account for how the different time periods and the distinct nature of each programme shaped their trajectories, we conceptualised a higher-order temporal macro-environmental dimension surrounding the programmes illustrating an evolving approach to digital transformation over time: from ambitions to create a novel centralised infrastructure (NPfIT), to advance digital maturity and learning across exemplar sites (GDE Programme), to validate and scale the uptake of new AI applications (AI Lab). The NPfIT emerged in the early 2000s, when large-scale centralised IT programmes were politically favoured, but the infrastructure, technology and local readiness were not mature, especially in hospital settings. The GDE Programme reflected a shift towards locally driven transformation, leveraging exemplars and blueprints at a time when digital maturity and organisational transformation were increasingly recognised as important, reflecting a shift towards the socio-organisational aspects of change and an increasing focus on local involvement in decision making, and learning from the experiences of NPfIT. The NHS AI Lab represented a further shift, focusing not on basic infrastructure and interoperability but on proving and upscaling the use of more advanced frontier technologies like AI, in an environment where digital systems were becoming more established, but the governance, ethics, and safe adoption were evolving. Over time, existing technologies and their applications became better understood and more mature, while new technologies and uses emerged.

We first applied the TPOM framework as a deductive coding structure to sensitise the analysis to established sociotechnical dimensions. TPOM codes were not mapped one-to-one to first-order concepts. Individual TPOM codes could contribute to more than one emerging concept. First-order concepts were developed through an interpretive process that considered combinations of TPOM codes and the relationships and tensions between these codes across the data, rather than through a direct correspondence between a single deductive code and a single concept. During coding, we also developed inductive codes to capture empirically salient issues that did not fit neatly within the TPOM categories. These constituted instances when data did not fit within the pre-existing TPOM codes. They mainly related to specific programme characteristics and interventions (e.g. the role of Local Service Providers who delivered national contracts in the NPfIT, the Blueprinting activity in GDE, or the ethics and regulatory work strand in the AI Lab evaluation).

**Fig. 2:**
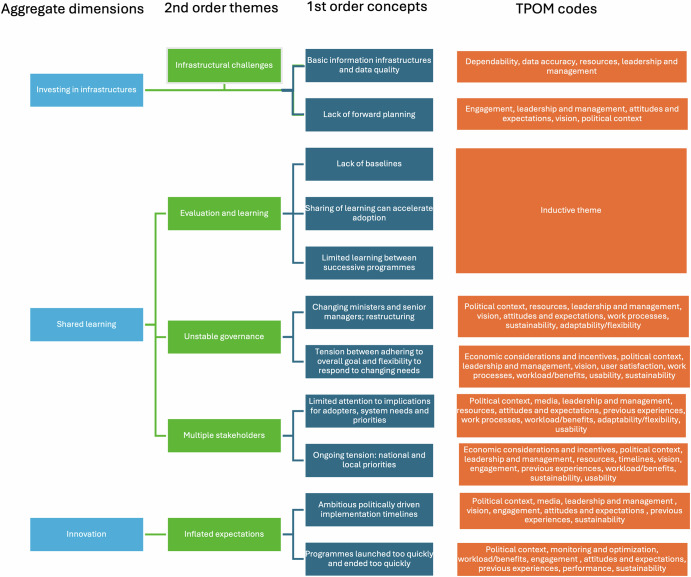
Mapping of TPOM codes, first-order concepts, second-order themes, and emerging aggregate dimensions (created with PowerPoint).

Through iterative comparison and refinement, these codes were examined both within and across TPOM dimensions, with particular attention to areas of tension, overlap, and recurring patterns across dimensions. This process informed the identification of the main analytical themes reported in the Results section, which reflect not only dimension-specific challenges but also the interactions between technological, human, organisational, and macro-level factors shaping implementation and adoption strategy. Supplementary Table 1 illustrates how identified themes mapped onto TPOM categories and also identifies new emergent categories. Emergent themes were identified because they included codes across all TPOM dimensions. For example “evaluation” as a theme was included because issues relating to evaluation and learning consistently emerged across cases as salient organisational processes shaping implementation, despite not constituting a distinct TPOM dimension. A mapping of first-order concepts (the TPOM and inductive codes), second-order themes (the themes presented in the Results section) and aggregate dimensions (the dimensions presented in the ILIAD model) is presented in Fig. 2^49^.

### Ethical approval

Ethical approval was obtained from the Research Ethics Committee in the University of Edinburgh School of Social and Political Science to undertake the Independent Evaluation of the GDE programme (reference number 4126 2017), and for the Independent Evaluation of the AI Lab (reference number 3228). The NHS CRS evaluation was reviewed by an ethics committee and classed as a service evaluation. All participants gave written informed consent to participate in the work.

## Supplementary information

Supplementary Table 1.

## Data Availability

The datasets generated and/or analysed during the current study are not publicly available due to ethical and confidentiality constraints but meta-data are available from the corresponding author on reasonable request.
