# Supplementary material for: Large-scale system-level digitalisation initiatives in the National Health Service in England: insights from three national evaluations
Source: NPJ Digit Med. 2026 Mar 2;9:301. doi: 10.1038/s41746-026-02495-8 (PMC13066607; doi:10.1038/s41746-026-02495-8)
Supplement: Supplementary file 1 — Supplementary Table 1. [file 41746_2026_2495_MOESM1_ESM.pdf]

Supplementary Table 1: Illustrative quotes for each of the factors from the various evaluations

Identified theme	TPOM dimension or inductively identified theme	National Programme for Information Technology	Global Digital Exemplar Programme	Artificial Intelligence Lab
**Infrastructural challenges**				
Basic information infrastructures (Wi-Fi, EHRs) and data quality crucial, but often an afterthought	Dependability<br>Data accuracy<br>Resources<br>Leadership and management	"I would argue strongly the opposite (Wi-Fi is needed) … It's something we would probably have put in the original business case if we didn't have it already, we'd put it in against other projects, to satisfy other objectives but we couldn't really be operating a clinical record in a ward environment without a proper, robust, secure wireless network." IT Manager	"And I think, we were already a number of steps on this process with the GDE programme, and I think that's really, really helped us. Because if we'd started from ground zero, like a number of [provider organisations] have tried to, it must've been remarkably tricky. We also already had quite a lot of the structure in place, quite a lot of the background sort of business intelligence, and things,	"The big, big thing that everybody who is close to the NHS is aware of, but nobody, it never really ever gets spoken about is that NHS data is there's a lot of it, but the quality of it is often quite poor. It actually needs a lot of work before it is useful." Strategist

			available, to be able to bolt on, really very rapidly, the resources that the clinicians need.” Clinical digital leader	
Lack of forward planning	Engagement<br>Leadership and management<br>Attitudes and expectations<br>Vision<br>Political context	“Our server room was not going to be big enough for the new system, the infrastructure they had here was not going to be big enough. We had to move to N3 connection, we didn’t have N3 connection at that time, so there was quite a lot of infrastructure work. And so what was happening, [software] was new and it was fragile, but also we had a server room that was fragile as well.” IT Manager	“I think as an IT department, the [provider organisation] has probably learnt that we need to consider how we’re putting systems in and not just to put systems in and launch them, so to consider all what’s needed, what are the benefits of putting it in, what will the benefits to the [provider organisation] be. So I think that has made us stop and think a bit more about that side of things, rather than just going out there and launching new systems and putting new pieces of kit into places.” GDE programme staff	“You've got little pockets of excellence and pockets where you've got champions who are really driving things forward and then huge lack of engagement elsewhere. And it's not. Sometimes it's lack of interest. But I think predominantly it's lack of time and energy and priority.” Manager

**Inflated expectations**				
Ambitious politically driven implementation timelines	Political context<br>Media<br>Leadership and management<br>Vision<br>Engagement<br>Attitudes and expectations<br>Previous experiences<br>Sustainability	“I think the problem is that because this has had such high-level sponsorship, I think it’s very difficult for people to step up. I think that from the top down there comes a very strong message that, you know, we’re not going to trash this. I mean, we’ve received a lot of adverse publicity, but I don’t particularly want to trash it. The difficulty is, as there’s a continuing failure to learn when mistakes are made, you just get more desperate about trying to sort things out.” Manager	“I think one of the main parts that was really effective is the pace-setting element of the GDE… The pace-setting as part of the programme was a massive part of achievements. And I think the reason for that is it really focuses the board. Because you have essentially money attached to a deadline to achieve something, that’s extremely motivating. And in [provider organisations] where you have so many competing priorities … I thought was very effective actually that we had to hit certain milestones with good quality and that then funding would be achieved. And I think that really helped focus the	“Well, our conclusion is that NHS England are beating up boards really badly, so you know, boards are actually being brutally beaten up. They're then passing on to their CIOs. So it's inexperienced leaders that that, you know, at organisational level. But there's definitely been a change of tone from NHS England… the Department of Health is driving some of that as well.” Strategist

			board. And because of that, we had a really, I think, strong functioning Digital Oversight Committee through the programme and that’s one of the things that kept the momentum going.” Clinical digital leader	
Programmes launched too quickly and ended too quickly	Political context<br>Monitoring and optimization<br>Workload/benefits<br>Engagement<br>Attitudes and expectations<br>Previous experiences<br>Performance<br>Sustainability	“The milestones in the plan were set as a contractual milestone, so we weren’t allowed to alter those. What was quite difficult was we had to work backwards from those milestones. … milestones that were set were probably going to be unachievable, but we had to work within the constraints of that contract.” IT Manager	“I think one of the dangers of GDE only funding a limited number of [provider organisations] is that you are just going to get very, very concentrated expertise and how is this going to ripple out to the wider NHS.” Clinical digital leader	“…NHSX 'cause they barely got out of the ground before the kind of pandemic kind of hit. But this was one of their kind of showpiece kind of, you know, flagship kind of programmes of work. I think it attracted quite a lot of funding quite quickly…You know that there was a flurry of announcements around the funding awards that that AI projects got.” Independent Sector

**Unstable governance, changing objectives and components over time**				
Changing ministers and senior managers; re-structuring of change management bodies	Political context<br>Resources<br>Leadership and management<br>Vision<br>Attitudes and expectations<br>Work processes<br>Sustainability<br>Adaptability/flexibility	"Well I think the jury's really out on it, the, it's interesting that the minister who's now got responsibility for NHS IT is new to the health field or at least, this is Simon Burns, rather he was in health earlier in his career but as I understand has had no, you know, involvement for some years. So to some extent the politicians who are quite vocal in this area such as Steven O'Brian the Conservative MP he's now gone off to, he's a minister in international development and Norman Lamb who was the Lib Dem health spokesman he's I believe the main policy advisor	"It's a multiagency programme and by their very nature there's always a little ambiguity in the governance, but our governance seems to have drifted in change with the changing responsibilities of different bodies during the lifetime of the Programme. And that's not unusual but it has been particularly disruptive I think on this programme. Even changes at the top of the shop, changes of Secretary of State has an impact on our programme, because the ultimate goal or importance that the Programme is given, changes with that change of	"The AI lab was part of NHSX before it came into NHS England and NHSX was always seen as much more innovative...they've come in, they've been scooped up and merged into NHS England and I think that's been very constraining actually...you hear people saying things feel much harder now, much more constrained, less kind of less experimental, less innovative."<br>NHS England

		to Nick Clegg so it's some new faces so what is the new government going to do, I think we wait to see." Independent Sector	direction." Engagement lead	
Tension between adhering to overall goal and flexibility to respond to changing needs	Economic considerations and incentives<br>Political context<br>Leadership and management<br>Vision<br>User satisfaction<br>Work processes<br>Workload/benefits<br>Usability<br>Sustainability	"The whole process of doing things which was built into the structure of the National Programme, I think, has been overly bureaucratic in an attempt to retain central control... What we are talking about now is the configuration of a solution. You start with a standard solution but you then configure the way it is presented and the way it works to meet the needs of a local hospital, as long as you don't do anything which alters its abilities to transmit information	"I think the other problem that we would describe with GDE, is that it has established a range of targets, so closed loop medicines administration. We are required to do that, to meet our GDE commitment, but that doesn't give any consideration as to whether we want to do that, or whether that would be a key priority for us at this moment in time. So, it is creating a tension between what we want to do and what we need to do." Non-clinical digital leader	"Interviewee If you try and control it through one structure, I think you are going to create bottlenecks and risk of failure. I think you will stifle innovation. So I think the theory of a new approach which is to create strong policy and hopefully adoption frameworks, but allowing you know substantial local activity within them within a real structure makes sense." Policy

		between other organisations." Supplier		
**Multiple stakeholders with conflicting agendas**				
Change is technology-driven with limited attention to implications for adopters, system needs and priorities	Political context<br>Media<br>Leadership and management<br>Resources<br>Attitudes and expectations<br>Previous experiences<br>Work processes<br>Workload/benefits<br>Adaptability/flexibility<br>Usability	"[supplier] have written [the system] with no clinical understanding. I would rather it has been written by a clinician. It's not a helpful system. It's too clumsy" Nurse	"I think pursuit of HIMSS has hindered us in our development. I think it's delayed some things that we wanted to do and could have done sooner because we've had to focus on HIMSS" Clinical digital leader	I think unfortunately the answer is very simple, which is even if you ask we both we all both we all work in academia which is just we and we ask these kinds of questions to students which is where do you start, you start with the problem with the clinical need and for whom. So rather than you start with a solution which is AI and who's regulating it, mm hmm to start with. So I think that's it's it's basic we just start there. Unfortunately, they what they did was to end up end there for them to discover that they were in some cases. Yeah, solving the wrong problems." Academic
Ongoing tension: national and local priorities	Economic considerations and incentives<br>Political context<br>Leadership and management<br>Resources<br>Timelines<br>Vision<br>Engagement<br>Previous experiences	"If you keep giving people the ability to localise things you kind of drive away from a centralised understanding, so it's a balancing act and I'm not sure whether you can ever meet everybody's	"I think pursuit of HIMSS has hindered us in our development. I think it's delayed some things that we wanted to do and could have done sooner because we've had to focus	"So as an example, when we were going through procurement, we've had. And we've had people on the call and we've turned around and they've said I've got this problem with procurement and then other people have jumped in and gone. Ohh, I'm running the procurement for this place.

	Workload/benefits<br>Sustainability<br>Usability	needs" Healthcare Professional	on HIMSS." Clinical digital leader	This is how we dealt with it, or this one, or when we had certain suppliers it would causing problems. We would try and deal with it essentially so that everyone had the same consistent approach rather than trying to do things differently. And so there is a if. If I had to think about how AI gets deployed, I would suggest it's probably it needs. It needs central coordination." NHS England
**Evaluation and learning**				
Limited by lack of baselines and consequent inability to assess outputs and impacts	Inductively identified	"I think an understanding of the real benefits was lacking and obviously with that, of course, goes a relative lack of clinical engagement. If people really don't see an obvious benefit, that's a problem" Healthcare Professional	"We've gone through the right processes and we are focusing on getting solid baseline data." Clinical digital leader	"There's a lots of shaded in mystery…But what I understand is that initial business case…sort of promised a lot, but it didn't lead into a set of creation of baseline metrics." NHS England
Evidence that sharing of learning can accelerate adoption	Inductively identified	"it's been helpful where people have gone through the implementation in	"We've only been able to do that because of the GDE and the reason being	"Blueprints around implementation so that if people want to do like a large scale trial like this, great, have at it. If you

		other trusts, because there have been lessons learned from other Trusts and they bring with them some knowledge” Psychiatric Consultant	the savings involved in working with other trusts and understanding pitfalls and not making the same mistakes over and over again, means we had the most rapid rollout of observations ever done for that company.” Clinical digital leader	want to go and do your own local thing, either based on direct learnings from this or is there more nebulous thing that you can draw on, say, if you're a district centre that doesn't have the expertise and the capacity to go and do this from scratch, you've got something to start from and you know, I should look, you've got a Bank of resources.” Evaluator
Limited learning between successive programmes	Inductively identified	“What needs to come out is a lot of people need to realise that there hasn’t been learning, that’s got to come out from this experience, and you know, if you deny that bad things happen, you don’t learn from them, and it’s that lack of openness around learning from what’s gone before which just keeps the NHS, well, there are big issues of the risk that	“I think there’s not much choice to be honest, was there, you know. These programmes are happening, these projects are moving forward, you need to engage. And certainly from that, you know, the feedback that I get from our staff across the Trust now is they want to be involved in implementing ...And, you know, we’ve had great engagement in putting the maternity EPR in, which again	“... if I have one criticism, I suppose of this stuff from the AI Lab...from their AI awards, where they spent 113 million, I don't think the transparency is there for the rest of the NHS to learn from those... where's the documentation from these awards? Where are the reports from the people doing these awards? ...I don't know what the answer is, but experience has taught me. If you ask for something and someone can't give it to you, it either doesn't exist or they're not being transparent because it doesn't say the thing that they wanted.” Programme Manager

		we’re exposed to by these things and nobody takes that the least seriously.” Manager	isn’t GDE but, however, followed a similar pattern to their other programmes of work.” Manager	
